# Deletion of *gltA* attenuates virulence and confers immune protection against *Salmonella* Enteritidis

**DOI:** 10.3389/fimmu.2026.1869123

**Published:** 2026-07-15

**Authors:** Siping Zhu, Miao Wang, Xintong Zhu, Jianghong Zhang, Zhixin Zhu, Guowang Chen, Chao Ren, Hong Li, Chihuan Li, Qiumei Shi, Zhiqiang Zhang

**Affiliations:** 1 Hebei Key Laboratory of Preventive Veterinary Medicine, Hebei Normal University of Science & Technology, Qinhuangdao, China; 2 Public Health Department, Wuhan Animal Disease Prevention and Control Center, Wuhan, China

**Keywords:** gene deletion, *gltA*, immune protection, *Salmonella* Enteritidis, virulence

## Abstract

**Background:**

*Salmonella enterica serovar Enteritidis* (*S*. Enteritidis) is a leading global cause of foodborne illnesses. The emergence of multidrug-resistant strains has exacerbated clinical treatment challenges and public health threats. The tricarboxylic acid (TCA) cycle is central to bacterial carbon metabolism, with key enzyme-encoding genes linked to environmental adaptation and pathogenicity. However, the biological role of Δ*gltA*, which encodes citrate synthase, the rate-limiting enzyme initiating the TCA cycle, in *S*. Enteritidis, and its potential as an attenuated vaccine target remain poorly understood.

**Methods:**

We constructed a *gltA* deletion mutant (Δ*gltA*) and its complemented strain (Δ*gltA*+*gltA*) from *S*. Enteritidis C50336of using λ-Red homologous recombination. We systematically characterized the effects of Δ*gltA* deletion on bacterial growth, antimicrobial susceptibility, and stress tolerance. Transcriptomics and qRT-PCR were employed to investigate virulence regulation mechanisms, while mouse models were used to evaluate attenuation and immunoprotective efficacy.

**Results:**

Δ*gltA* deletion did not alter growth in nutrient-rich LB or nutrient-poor M9 media, but significantly impaired motility and biofilm formation by inhibiting curli fimbriae and cellulose synthesis, and increased susceptibility to 15 antimicrobial agents. The Δ*gltA* mutant also showed reduced tolerance to acidic, alkaline, oxidative, thermal, and osmotic stresses. Notably, intraperitoneal infection revealed >10^6^-fold attenuation in Kunming mice (wild-type LD_50_ = 3.16 × 10³ CFU; no mortality at 1 × 10^9^ CFU Δ*gltA*), with decreased organ bacterial loads and global downregulation of 24 key virulence genes. A single oral immunization with Δ*gltA* elicited robust humoral and cellular immunity, conferring promising protection against lethal wild-type challenge.

**Conclusion:**

Deletion of Δ*gltA* is associated with altered metabolic homeostasis, impaired environmental adaptability and attenuated pathogenicity in *S*. Enteritidis. The Δ*gltA* mutant conferred effective protective immunity in the mouse model used here, serving as a promising candidate strain and molecular target for further development of live attenuated *S*. Enteritidis vaccines.

## 1 Introduction

*Salmonella* is the leading bacterial cause of foodborne diseases worldwide. It causes millions of infections and significant mortality each year, posing a major global public health threat. *Salmonella enterica* serovar Enteritidis (*S*. Enteritidis) is one of the most prevalent serotypes currently causing human salmonellosis (1–3). As a prototypical zoonotic pathogen, *S*. Enteritidis primarily colonizes poultry and livestock as reservoir hosts (4). It enters the food chain through contaminated eggs, meat, and dairy products, and is a major cause of global foodborne outbreaks. In humans, mild *S*. Enteritidis infection typically manifests as self-limiting gastroenteritis, while severe infection can progress to systemic complications including sepsis, meningitis and arthritis, with disproportionately high mortality observed in infants, the elderly and immunocompromised individuals. In livestock production, *S*. Enteritidis infection causes high mortality in young animals, asymptomatic carriage and vertical transmission in adult animals, resulting in enormous economic losses while serving as a persistent source of human infection. In recent years, inappropriate antimicrobial use has driven the widespread spread of multidrug-resistant *S*. Enteritidis strains with expanding resistance profiles, significantly complicating clinical treatment (5, 6). The horizontal transfer of resistance genes through the food chain further exacerbates public health risks. Therefore, developing safe and effective strategies for *S*. Enteritidis prevention and control represents a critical unmet need in veterinary public health.

Vaccination is an economical and effective strategy for controlling bacterial infectious diseases, and also a core approach to preventing the spread of bacterial resistance. Inactivated vaccines have limitations such as a single immune pathway, the ability to induce only humoral immunity, a short duration of protection, and the need for multiple booster doses; furthermore, endotoxins are difficult to remove. Subunit vaccines, on the other hand, suffer from issues such as low immunogenicity, high production costs, and poor ability to induce mucosal immunity (7). Neither vaccine type meets the dual needs of large-scale livestock production and human public health protection. By contrast, live attenuated vaccines can be administered via oral routes, mimicking natural infection, simultaneously activating humoral, cellular, and mucosal immunity to confer comprehensive and long-lasting protection, making them the primary focus of *Salmonella* vaccine development. The key to developing a live attenuated vaccine lies in identifying safe and effective targets for attenuation. An ideal target must meet two core criteria (8, 9). First, the deletion of the target gene significantly attenuates virulence without risk of reversion, ensuring biosafety. Second, deletion does not impair bacterial growth or immunogenicity, enabling robust induction of protective immune responses. Currently reported attenuation targets for Salmonella are primarily genes related to amino acid synthesis (such as *aroA*) and cyclic adenosine monophosphate receptor proteins (*crp*). However, some attenuated strains exhibit defects such as residual virulence, reduced immunogenicity, and weak ability to colonize the host. Therefore, screening for novel, safe, and highly effective attenuation targets remains a top priority for *S*. Enteritidis live attenuated vaccine development (10).

Bacterial central carbon metabolism is essential for survival and proliferation in both the external environment and host organisms. The tricarboxylic acid (TCA) cycle is the central hub of carbon and energy metabolism, providing ATP and key metabolic intermediates for bacterial life processes. Furthermore, it is also closely linked to environmental adaptability, host colonization, and pathogenicity. Accumulating evidence shows that deletion of genes encoding key TCA cycle rate-limiting enzymes significantly impairs stress tolerance, intracellular survival, and virulence in diverse pathogens, such as *Mycobacterium tuberculosis* (11), *Vibrio cholerae* (12), and *Salmonella spp* (13, 14), making the TCA cycle a promising source of attenuation targets. The *gltA* gene encodes citrate synthase, the first rate-limiting enzyme of the TCA cycle. It catalyzes the condensation of acetyl-CoA and oxaloacetate to form citrate, directly determining TCA cycle flux and metabolic homeostasis. In *M. tuberculosis*, *gltA* deletion leads to altered T3SS expression and a significant decrease in virulence (15); in *Klebsiella pneumoniae*, functional defects in citrate synthase significantly reduce the strain’s organ fitness, affecting the pathogenic process (16); in avian pathogenic *Escherichia coli*, deletion of the *gltA* gene impairs the strain’s biofilm formation ability (18). However, to date, the biological function of the GltA in *S*. Enteritidis has not been systematically elucidated, and its impact on the infection and pathogenicity of *S*. Enteritidis remains unclear.

This study aimed to elucidate the role of citrate synthase GltA in the pathogenesis of *S*. Enteritidis. Using λ-Red homologous recombination technology, we constructed a *gltA* knockout strain (Δ*gltA*) and its complementation strain (Δ*gltA*+*gltA*) of *S*. Enteritidis strain C50336. By comparing the differences in growth characteristics, motility, biofilm formation, resistance to environmental stress, and virulence gene expression among the wild-type, knockout, and restorer strains, we comprehensively assessed the impact of *gltA* gene deletion on the bacterial biological properties. Furthermore, the median lethal dose (LD_50_) and *in vivo* bacterial load will be determined using a mouse infection model, and the efficacy of the induced immune protection will be further evaluated. Through these studies, the central regulatory role of the *gltA* gene in the biological characteristics and virulence of *S*. Enteritidis will be elucidated, and the molecular mechanisms underlying the association between metabolic regulation and virulence in *S*. Enteritidis will be refined.

## 2 Materials and methods

### 2.1 Bacterial strains and plasmids

*Salmonella enterica* serovar Enteritidis strain C50336 was isolated from a diarrheal patient’s fecal sample. It was purchased from the National Institutes for Food and Drug Control (NIFDC) of China. The strain was stored at the Hebei Key Laboratory of Preventive Veterinary Medicine. All strains were cultured in LB medium (Qingdao Haibo Biotechnology Co., Ltd.) at 37 °C. Ampicillin (100 μg/mL) or chloramphenicol (34 μg/mL) was added when required. Plasmids pKD3, pKD46, pBR322, and pCP20 for gene knockout were obtained from Invitrogen. The pMD-19T vector was purchased from Baoriyi Biotechnology Co., Ltd (Beijing, China).

### 2.2 Experimental animals

Female Kunming mice (6–8 weeks old) were purchased from Beijing Sipeifu Biotechnology Co., Ltd. (Beijing, China).

### 2.3 Construction of *gltA* deletion mutant and complemented strain

The Δ*gltA* gene was deleted using λ-Red homologous recombination. First, the *cat* gene was amplified from plasmid pKD3 with primers P1 and P2 (**Table 1**). The purified PCR product was electroporated into C50336 competent cells harboring pKD46. The recombinant strain Δ*gltA*::*cat* was obtained. Plasmid pCP20 was then electroporated to remove the inserted *cat* gene. The resulting strain was incubated at 42 °C for 5–6 h to eliminate the temperature-sensitive pCP20 plasmid. The Δ*gltA* deletion was verified by PCR with primers P3 and P4 (**Table 1**). The purified PCR product was cloned into the pMD-19T vector (Takara Biomedical Technology, Beijing). The recombinant plasmid was sequenced by Sangon Biotech (Shanghai) Co., Ltd. The knockout of Δ*gltA* gene was further confirmed by detecting gene expression by qPCR. The confirmed strain was designated C50336 Δ*gltA*.

**Table 1:** PCR primer information.

Primers	Nucleotide sequences (5′-3′)
P1(Δ*gltA*F)	CCGACGCAGGAAGAGTATGACGAGTTCAGAA CGACAGTCACCCGCCATACGATGATCCA TGTGTAGGCTGGAGCTGCTTCG
P2(Δ*gltA*R)	TCACGGTGAACATGGAGGACGGAATGCCCA TCGCTTTCAGAATGATGCCGGAGTAGAAG CATATGAATATCCTCCTTAG
P3(ID-*gltA*F)	CGCTAAGGAGACCGTAAATGG
P4(ID-*gltA*R)	CGTACTACCTCGCAAACTCAACAC
P5(Δ*gltA*+*gltA*F)	ATGATATTGACCCATTCTTTCAGG
P6(Δ*gltA*+*gltA*R)	TTAACGCTTCAGCGCCGATTTA
P7 (*gltAF)*	TACCACGACTCGCTGGATG
P8 (*gltAR)*	CGGATAAACAAACGGCTGA

To construct the complemented strain, the full-length Δ*gltA* open reading frame was amplified from C50336 genomic DNA with primers P5 and P6 (**Table 1**). The amplicon was cloned into plasmid pBR322 to generate pBR322-*gltA*. The recombinant plasmid was electroporated into Δ*gltA*. Positive clones were verified by PCR with primers P3 and P4. The complemented strain was named Δ*gltA*+*gltA*. *gltA* expression in Δ*gltA* and Δ*gltA*+*gltA* was confirmed by qPCR. Total RNA was extracted using a Bacterial RNA Extraction Kit (Beijing Aidlab Biotechnologies Co., Ltd., RN63). cDNA was synthesized by reverse transcription. qPCR was performed with primers P7 and P8 (**Table 1**) to assess Δ*gltA* expression levels.

### 2.4 Growth curve analysis

Overnight bacterial cultures were diluted 1:100 into fresh Luria-Bertani (LB) medium and M9 minimal medium. Cultures were incubated at 37 °C with shaking at 180 rpm. The OD_600_ value of the bacterial culture was measured every hour until all strains reached the stationary phase. Growth curves were plotted to analyze bacterial growth characteristics (19).

### 2.5 Bacterial motility assay

The motility of each strain was assessed by measuring the zone of growth on semi-solid medium. A 5 µL aliquot of the bacterial culture of each strain was inoculated onto LB semi-solid agar plates containing 0.3% agar and incubated in a 37 °C incubator for 5–6 hours. Bacterial motility was evaluated by measuring the diameter of the growth zone formed on the semi-solid agar plates (20).

### 2.6 Biofilm formation assay

Biofilm formation was detected and quantified by crystal violet staining (20). Overnight cultures of C50336, Δ*gltA*, and Δ*gltA*+*gltA* were diluted 1:100 into 6 mL LB medium in glass tubes. Tubes were incubated statically at 30 °C for 3 days. The supernatant was discarded. Tubes were washed 2–3 times with PBS. Biofilms were fixed with anhydrous methanol for 15 min and stained with 2% crystal violet for 15 min. Biofilm formation was assessed by observing the stained ring on the tube wall.

Quantitative analysis of biofilm formation was performed using a 96-well plate method (20). Briefly, 150 μL of bacterial suspension was added to each well and stained as described above. After staining, 200 μL of anhydrous ethanol was added to each well. Absorbance was measured at 570 nm. All experiments were performed in triplicate.

Curli fimbriae and cellulose, the two major biofilm components, were detected separately (20). A 5 μL aliquot of each bacterial suspension was spotted onto salt-free LB agar plates containing 60 mg/L Congo red and 10 mg/L Coomassie brilliant blue (for Curli detection) or 200 mg/L Calcofluor white (for cellulose detection). Plates were incubated statically at 28 °C for 2 days, and colony color, morphology, and fluorescence intensity under 366 nm ultraviolet light were observed.

### 2.7 Determination of minimum inhibitory concentrations

For this study, 15 antimicrobial agents were selected (including ceftriaxone, cefradine, spectinomycin, neomycin, amikacin, kanamycin, norfloxacin, ciprofloxacin, enrofloxacin, doxycycline, florfenicol, sulfamethoxypyridazine, bacitracin, tilmicosin, and acetylmethoxypyridine, all purchased from Shanghai Yuanye Biotechnology Co., Ltd.) for MIC testing. Log-phase bacteria were adjusted to a concentration of 1 × 10^6^ CFU/mL, and a two-fold serial dilution of the antibiotics was performed using hydrolyzed casein (MH) medium. Equal volumes of the bacterial suspension were mixed with the antibiotic solutions at different dilutions, and the mixtures were incubated in a 37 °C incubator for 16–18 hours. *E. coli* ATCC 25922 was used as the control strain, and the experiment was repeated three times (21).

### 2.8 Environmental stress resistance assays

The tolerance of bacteria to environmental stress was evaluated by measuring the survival of each strain under mimicked stress conditions (22). Briefly, bacteria were cultured to the mid-logarithmic phase and diluted to 1×10^7^ CFU/mL in saline. Bacterial suspensions were diluted 1:100 into the following stress conditions and incubated for 1 h under acidic stress (pH 4.0), alkaline stress (pH 10.0), heat stress (42 °C), hyperosmotic stress (2.5 mol/L NaCl), and hypoosmotic stress (deionized water). For oxidative stress, bacteria were treated with 10 mmol/L H_2_O_2_ in PBS for 10 min. After treatment, bacteria were serially diluted in PBS (Thermo Fisher Scientific, China) for plate counting. Survival rates were calculated as the ratio of colony counts after treatment to those before treatment.

### 2.9 Median lethal dose determination in mice

Fifty-five mice were randomly divided into 11 groups (n=5 per group). Five groups were intraperitoneally injected with C50336 at doses ranging from 1×10^5^ to 1×10^9^ CFU per mouse. Another five groups were injected with Δ*gltA* at the same dose range. The control group received an equal volume of PBS intraperitoneally. Mice were monitored daily for abnormal behavior and mortality for 14 days. LD_50_ values were calculated using Karber’s method (22).

### 2.10 Bacterial loads in organs

Twenty mice were randomly divided into two groups (n=10 per group). Mice were inoculated with wild-type C50336 or Δ*gltA* at 1×10^4^ CFU per mouse. Liver and spleen tissues were collected aseptically at 7, 14, 21, and 28 days post-infection. Tissues were weighed and homogenized in sterile PBS. Homogenates were serially diluted and plated for colony counting. Bacterial loads in the liver and spleen were recorded at each time point (22).

### 2.11 RNA extraction and quantitative real-time PCR

Total RNA was extracted using a bacterial RNA Extraction Kit (Beijing Aidlab Biotechnologies Co., Ltd.). Genomic DNA was removed using DNase I (Aidlab). cDNA was synthesized using a Reverse Transcription Kit (Bohang Biotechnology Co., Ltd.). qRT-PCR was performed using SYBR^®^ PreMix Ex Taq II (Takara Biotechnology, Dalian). Relative gene expression levels were calculated using the 2-ΔΔCt method. Expression levels of 24 key virulence genes were normalized to the 16S rRNA gene and compared to those in C50336. Primer sequences for qRT-PCR are listed in **Table 2**.

**Table 2:** qPCR primer information.

Primers	Nucleotide sequences (5′-3′)
fimDF	CGCGGCGAAAGTTATTTCAA
fimDR	CCACGGACGCGGTATCC
flgGF	GCGCCGGACGATTGC
flgGR	CCGGGCTGGAAAGCATT
hflKF	AGCGCGGCGTTGTGA
hflKR	TCAGACCTGGCTCTACCAGATG
invHF	CCCTTCCTCCGTGAGCAAA
invHR	TGGCCAGTTGCTCTTTCTGA
lrpF	TTAATGCCGCCGTGCAA
lrpR	GCCGGAAACCAAATGACACT
mrr1F	CCATCGCTTCCAGCAACTG
mrr1R	TCTCTACCATGAACCCGTACAAATT
ompRF	TGTGCCGGATCTTCTTCCA
ompRR	CTCCATCGACGTCCAGATCTC
orf245F	CAGGGTAATATCGATGTGGACTACA
orf245R	GCGGTATGTGGAAAACGAGTTT
pipBF	GCTCCTGTTAATGATTTCGCTAAAG
pipBR	GCTCAGACTTAACTGACACCAAACTAA
prot6EF	GAACGTTTGGCTGCCTATGG
prot6ER	CGCAGTGACTGGCATCAAGA
rfbHF	ACGGTCGGTATTTGTCAACTCA
rfbHR	TCGCCAACCGTATTTTGCTAA
sipAF	CAGGGAACGGTGTGGAGGTA
sipAR	AGACGTTTTTGGGTGTGATACGT
sipBF	GCCACTGCTGAATCTGATCCA
sipBR	CGAGGCGCTTGCTGATTT
sodCF	CACATGGATCATGAGCGCTTT
sodCR	CTGCGCCGCGTCTGA
ssaVF	GCGCGATACGGACATATTCTG
ssaVR	TGGGCGCCACGTGAA
ssrAF	CGAGTATGGCTGGATCAAAACA
ssrAR	TGTACGTATTTTTTGCGGGATGT
tatAF	AGTATTTGGCAGTTGTTGATTGTTG
tatAR	ACCGATGGAACCGAGTTTTTT
xthAF	CGCCCGTCCCCATCA
xthAR	CACATCGGGCTGGTGTTTT
mgtCF	CGAACCTCGCTTTCATCTTCTT
mgtCR	CCGCCGAGGGAGAAAAAC
spvBF	TGGGTGGGCAACAGCAA
spvBR	GCAGGATGCCGTTACTGTCA
csgDF	GCCTCATATTAACGGCGTG
csgDR	AGCGGTAATTTCCTGAGTGC
csgAF	AATGCCACCATCGACCAGTG
csgAR	CAAAACCAACCTGACGCACC
bcsAF	GCCCAGCTTCAGAATATCCA
bcsAR	TGGAAGGGCAGAAAGTGAAT
16SF	CCAGGGCTACACACGTGCTA
16SR	TCTCGCGAGGTCGCTTCT

### 2.12 Transcriptome analysis

C50336 and Δ*gltA* colonies were inoculated into LB liquid medium and cultured with shaking until OD_600_ reached 1.0–2.0. Bacteria were washed three times with sterile PBS. Bacteria pellets were immediately frozen in liquid nitrogen and stored at -80 °C. Total RNA was isolated from frozen bacterial samples. RNA sequencing and bioinformatics analysis were performed by Shanghai Majorbio Bio-Pharm Technology Co., Ltd. using the Illumina platform. The specific steps are as follows: RNA purity, integrity and concentration were rigorously evaluated using a Nanodrop spectrophotometer and Agilent 2100 Bioanalyzer to eliminate samples with degradation or genomic DNA contamination. Qualified mRNA was enriched using Oligo (dT) magnetic beads and randomly fragmented for subsequent library construction. Strand-specific cDNA libraries were generated via reverse transcription, terminal repair, 3′-end A-tailing, sequencing adapter ligation and PCR amplification. After strict library quality verification, high-quality libraries were subjected to paired-end 150 bp (PE150) sequencing on the Illumina NovaSeq platform. For bioinformatics analysis, raw sequencing reads were processed and quality-filtered using Fastp software to remove low-quality reads, adapter sequences and short fragments, yielding high-quality clean reads. The clean reads were precisely mapped to the reference genome of *S*. Enteritidis for gene localization and expression quantification. Differentially expressed genes (DEGs) between the Δ*gltA* mutant and C50336 were defined with a threshold of fold change > 2 or < 0.5 and adjusted P-value (padj) < 0.05. Furthermore, GO functional annotation and KEGG pathway enrichment analyses were conducted to systematically characterize the biological functions and enriched signaling pathways of the screened DEGs.

### 2.13 Immunoprotective efficacy assays

#### 2.13.1 Immunization and challenge protocol

Forty Kunming mice were randomly divided into two groups (n=20 per group). The immunization group was orally administered 1.0×10^7^ CFU of Δ*gltA*. The control group received an equal volume of PBS orally. Mice were monitored daily for survival and clinical signs. At 28 days post-immunization, both groups were challenged with 1.58×10^4^ CFU of C50336 via intraperitoneal injection. Mortality was recorded daily for 14 days post-challenge (22). The relative protection rate (RPS) was calculated as: RPS = [(number of dead mice in control group − number of dead mice in immunization group)/number of dead mice in control group] × 100%.

#### 2.13.2 Serum specific IgG and sIgA detection

Serum anti-*S*. Enteritidis IgG levels were measured by indirect ELISA (23). C50336 was cultured to mid-logarithmic phase. Bacteria were lysed by sonication and centrifuged. The supernatant was used as the coating antigen (1 μg per well). Incubate the culture plates at 37 °C for 1 h, then incubate overnight at 4 °C. The next day, wash the culture plates five times with PBS containing 0.5% Tween (PBST), and block them for 2 h at 37 °C with PBS containing 5% skim milk. Blood samples were collected from the tail vein of three mice per group at 0, 7, 14, 21, and 28 days post-immunization. After washing the culture plate five times with PBST, add mouse serum as the primary antibody (diluted 1:200) to the reaction mixture and incubate at 37 °C for 1 h. The plate was then washed five times with PBST, and HRP-conjugated secondary antibody (1:10,000 dilution) was added, followed by incubation at 37 °C for 1 h. After five washes with PBST, 100 μL of TMB substrate solution (Beijing Solabo Technology Co., Ltd.) was added to each well, and the plate was incubated for 10 min at 37 °C in the dark. Finally, add 50 μL of 2 M sulfuric acid to each well to stop the reaction, and measure the OD450 value within 15 min.

On days 0, 7, 14, 21 and 28 post-immunization, three mice were chosen from each group. Mouse feces were collected and suspended in 500 μL PBS (pH 7.6) containing 100 μg/mL soybean trypsin inhibitor (Sigma), 10 mg/mL BSA (Sigma), and 30 mM disodium EDTA. After centrifugation at 4 °C, the supernatant was used as the primary sample. HRP-conjugated goat anti-mouse sIgA antibody served as the secondary antibody. All other procedures were consistent with those applied for IgG ELISA detection.

#### 2.13.3 Spleen index determination

Three mice per group were weighed at 7, 14, 21, and 28 days post-immunization. Mice were humanely euthanized by gradual CO_2_ inhalation (displacement rate 30%–40% chamber volume per minute) in accordance with AVMA guidelines. Mice were euthanized, and spleens were excised and weighed (23). The spleen index was calculated as (spleen weight/body weight) × 100%.

#### 2.13.4 Lymphocyte proliferation assay

Three mice per group were euthanized at 14 days post-immunization. The method of euthanasia is the same as in 2.13.3. Spleens were aseptically removed and homogenized. Splenocytes were isolated by filtration through a 70 μm cell strainer. Red blood cells were lysed using Red Blood Cell Lysis Buffer (Beijing Solarbio Science & Technology Co., Ltd.). Splenocytes were resuspended in RPMI 1640 medium (Thermo Fisher Scientific) supplemented with 10% FBS, 50 μg/mL penicillin, and 50 μg/mL streptomycin. Cell viability was assessed by trypan blue staining. Cells were counted using a hemocytometer. Splenocytes were seeded into 96-well plates at 5×10^5^ cells per well. Cells were stimulated with equal volumes of bacterial supernatant antigen (7.5 μg/mL), concanavalin A (ConA, 2 μg/mL), or PBS. Plates were incubated at 37 °C in 5% CO_2_ for 72 h. Lymphocyte proliferation was measured using an MTT Cell Proliferation and Cytotoxicity Assay Kit (Shanghai Beyotime Biotechnology Co., Ltd.) according to the manufacturer’s instructions (23). The stimulation index (SI) was calculated as: SI = (OD_450_ of stimulated group − OD_450_ of medium-only group)/(OD_450_ of unstimulated group − OD_450_ of medium-only group).

### 2.14 Ethics statement

All animal experiments were conducted in full compliance with international ethical standards and the Experimental Animal Regulation Ordinances (HPDST 2020-17) as stipulated by the Hebei Provincial Department of Science and Technology. The study protocol was reviewed and approved by the Animal Care and Use Committee of Hebei Normal University of Science and Technology.

### 2.15 Statistical analysis

Statistical analyses were performed using GraphPad Prism version 9.5.0. All quantitative data are expressed as mean ± standard error of the mean (SEM). Differences between the wild-type and mutant group, or between the immune group and PBS control group, were evaluated using unpaired two-tailed Student’s t-test. A value of P < 0.05 was considered statistically significant. Statistical significance was denoted as follows: p < 0.05, *p < 0.01, **p < 0.001.

## 3 Results

### 3.1 *The gltA* deletion does not alter *S.* Enteritidis growth

**Figure 1:**
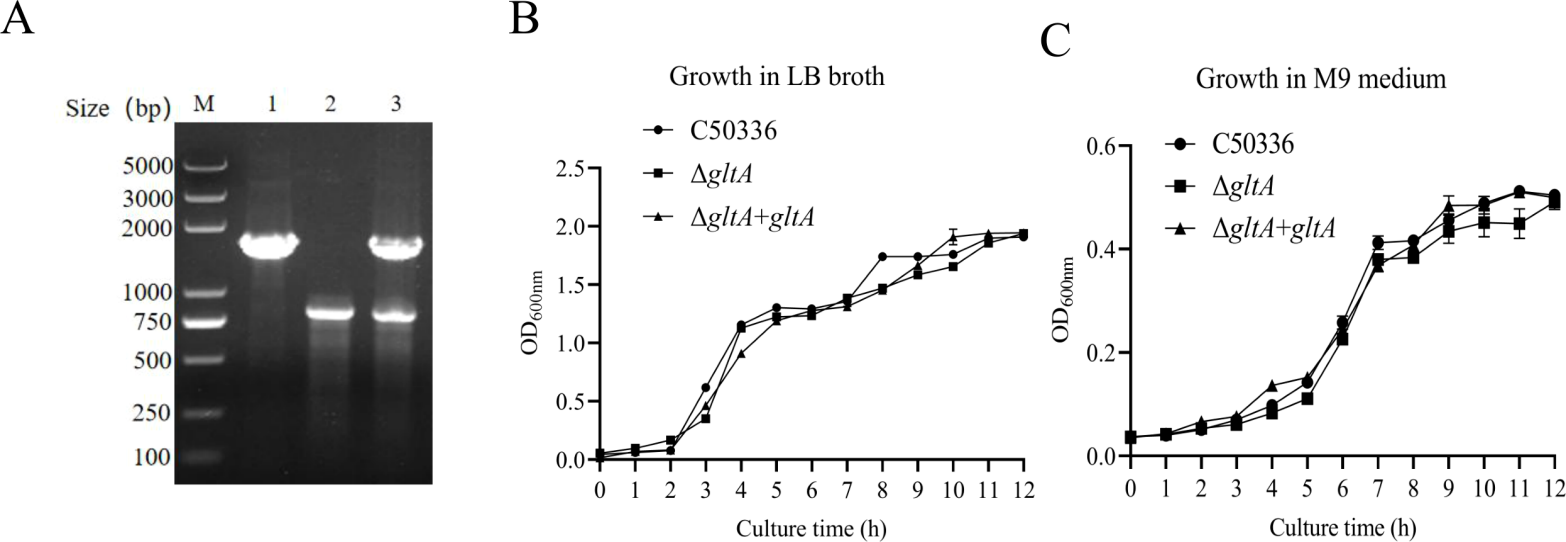
Construction of the Δ*gltA* mutant and growth curve analysis. **(A)** The Δ*gltA* gene was knocked out via λ-Red homologous recombination using pKD3, pKD46 and pCP20 plasmids. The complemented strain was generated by transforming recombinant pBR322-gltA into Δ*gltA*. Gene deletions and complementation effects were verified by PCR. **(B)** Strains were cultured in LB medium at 37 °C, 180 rpm shaking. OD_600_ was detected hourly to record proliferative traits. **(C)** Strains were cultured in M9 minimal medium at 37 °C, 180 rpm shaking. OD_600_ was detected hourly to record proliferative traits. Data are presented as mean values of three replicates.

The *gltA* deletion mutant (C50336 Δ*gltA*) and the complemented strain (Δ*gltA*+*gltA*) were generated via λ-Red homologous recombination. PCR analysis using flanking primers for the *gltA* gene revealed that the amplified fragments from the wild-type strain and the knockout strain were 1,500 bp and 900 bp, respectively, confirming the successful deletion of the *gltA* gene. In the complemented strain, we detected both sizes of fragments, the wild-type and the knockout fragment, confirming the successful construction of the complemented strain (**Figure 1A**). Additionally, qPCR and gene sequencing were employed to further validate the deletion and complementation of the *gltA* gene (data not shown).

Growth profiles were determined in LB and M9 media. No significant differences in growth were observed among C50336, Δ*gltA*, and Δ*gltA*+*gltA* (**Figures 1B, C**). These data demonstrate that Δ*gltA* deletion does not affect *S*. Enteritidis proliferation in either nutrient-rich or minimal media.

### 3.2 The *gltA* deletion impairs *S*. Enteritidis motility

**Figure 2:**
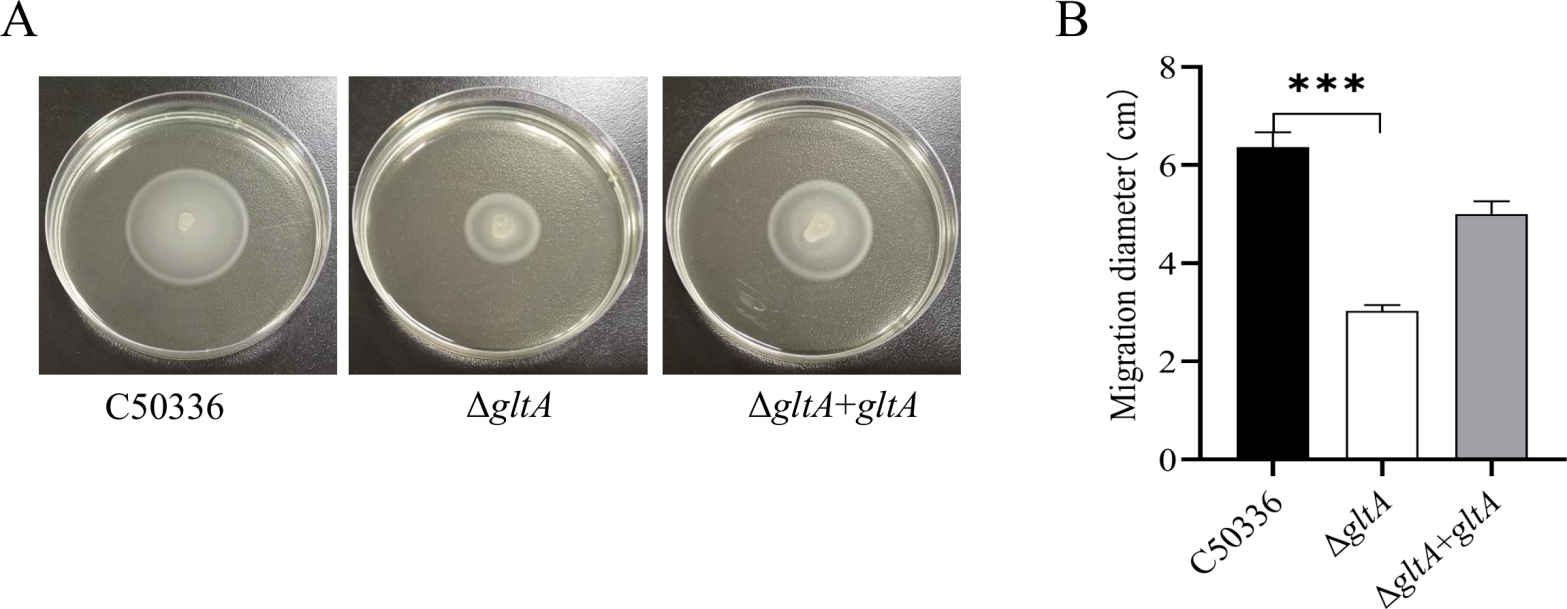
Motility assay. **(A)** The 5 μL bacterial suspension was spotted onto 0.3% semi-solid LB agar, incubated at 37 °C for 5–6 (h) Motility was quantified by measuring diffusion diameter of bacterial growth zone. **(B)** Quantitative results of bacterial motility analysis. Data are presented as mean values of three replicates. Data are presented as mean ± standard error of the mean (SEM) from three independent experiments. Statistical significance was determined by Student’s t-test for comparisons between two groups. (***p < 0.001).

Bacterial motility was evaluated on 0.3% semi-solid agar. The wild-type C50336 formed large, clear motility halos, indicating robust motility. In contrast, Δ*gltA* exhibited a significantly reduced motility diameter. Motility was partly restored to wild-type levels in the complemented strain (**Figures 2A, B**). These results indicate that loss of *gltA* correlates with severely impaired motility of *S*. Enteritidis.

### 3.3 The *gltA* deletion attenuates *S*. Enteritidis biofilm formation

**Figure 3:**
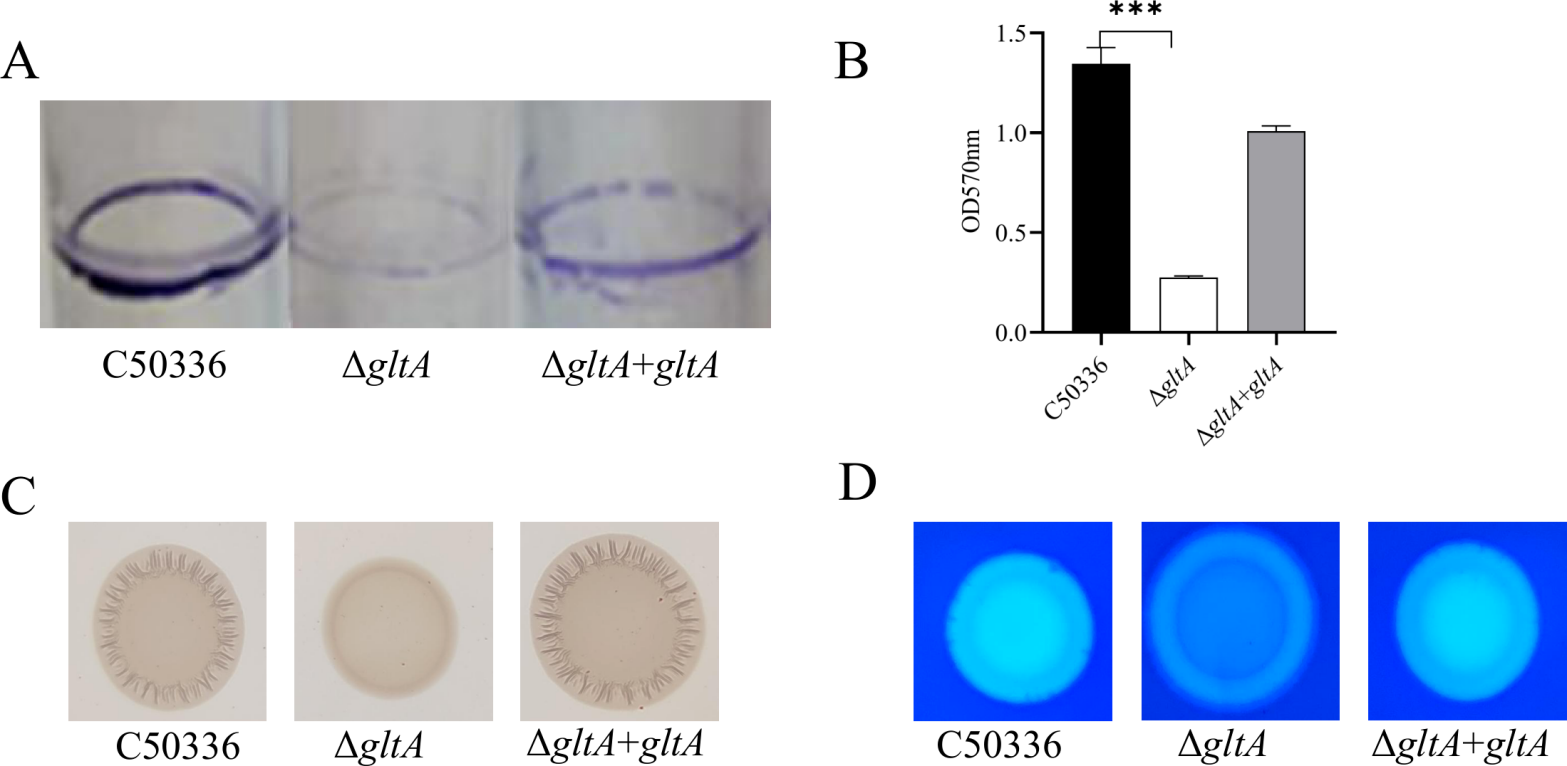
Analysis of biofilm formation and composition. **(A)** Qualitative assessment of biofilm assembly in glass tubes. Overnight bacterial cultures were diluted 1:100 in LB medium and incubated statically at 30 °C for 3 days. Culture supernatants were discarded, tubes washed 2–3 times with PBS, fixed with anhydrous methanol for 15 min, and stained with 2% crystal violet for 15 min to visualize adherent biofilm rings on tube walls. **(B)** Quantitative measurement of biofilm formation. Log-phase bacterial cultures of each strain were inoculated into a 96-well microplate and incubated for 3 days. The biofilms adhering to the well walls were stained with a 2% crystal violet solution, followed by triple washing with PBS. The crystal violet was then dissolved in methanol, and the absorbance was measured at 570 nm (***p < 0.001). **(C)** Colony morphology on Congo red agar. Each strain was inoculated onto Congo red agar containing 160 mg/L Congo red and 10 mg/L Coomassie Blue and incubated at 28 °C for 48 hours. The formation of curli fimbriae in the colonies of each strain was observed. **(D)** Fluorescence intensity analysis of colonies on Calcofluor white agar. Each strain was inoculated into medium containing a fluorescent whitening agent (200 mg/L), cultured at 28 °C for 48 hours, and the fluorescence intensity of the colonies was observed under UV light at 366 nm. These are representative images from replicate experiments. Data are presented as mean ± standard error of the mean (SEM) from three independent experiments. Statistical significance was determined by Student’s t-test for comparisons between two groups. (***p < 0.001).

Biofilm formation was assessed by crystal violet staining. C50336 formed thick, intensely stained biofilm rings on glass tubes, whereas Δ*gltA* produced thin, faint rings with drastically reduced biomass. Biofilm formation was partially restored in Δ*gltA*+*gltA* (**Figure 3A**). Quantitative 96-well plate assays confirmed that the OD_580_ value of Δ*gltA* was significantly lower than that of C50336 (**Figure 3B**).

Congo red and Calcofluor white staining were performed to analyze biofilm matrix components. C50336 and Δ*gltA*+*gltA* strains exhibited robust peripheral colony rugosity, whereas the Δ*gltA* mutant displayed smooth colony edges, indicating suppressed curli fimbriae production in Δ*gltA* (**Figure 3C**). Additionally, Δ*gltA* showed markedly reduced fluorescence intensity on Calcofluor white agar, with fluorescence restored in the complemented strain (**Figure 3D**). Collectively, these findings reveal that Δ*gltA* deletion is associated with suppressed production of curli fimbriae and cellulose, leading to reduced biofilm formation capacity.

### 3.4 The *gltA* deletion modulates *S*. Enteritidis antimicrobial susceptibility

Antimicrobial susceptibility was determined using the Kirby-Bauer disk diffusion method (**Table 3**). As shown in **Table 3**, the deletion of the *gltA* gene resulted in significantly increased susceptibility of *S*. Enteritidis to various antibiotics. Compared with the C50336 strain, the Δ*gltA* strain exhibited larger inhibition zones against various antibiotics, while reintroduction of the *gltA* gene partly abolished the increased antibiotic sensitivity. These results indicate that the *gltA* gene contributes to antibiotic resistance in *S*. Enteritidis.

**Table 3:** Antimicrobial susceptibility profiles of the *gltA* mutant.

No.	Class	Agent	Inhibition zone diameter (mm)		
			C50336	ΔgltA	ΔgltA+gltA
1	β-lactams	Ceftriaxone	30 ± 0.4	36 ± 0.5	32 ± 0.4
2	β-lactams	Cefradine	16 ± 0.3	28 ± 0.6	18 ± 0.3
3	Aminoglycosides	Spectinomycin	18 ± 0.3	24 ± 0.4	20 ± 0.3
4	Aminoglycosides	Neomycin	16 ± 0.2	20 ± 0.3	18 ± 0.2
5	Aminoglycosides	Amikacin	18 ± 0.3	20 ± 0.3	18 ± 0.3
6	Aminoglycosides	Kanamycin	16 ± 0.2	24 ± 0.5	16 ± 0.2
7	Quinolones	Norfloxacin	22 ± 0.4	30 ± 0.5	28 ± 0.4
8	Quinolones	Ciprofloxacin	28 ± 0.4	32 ± 0.4	30 ± 0.4
9	Quinolones	Enrofloxacin	22 ± 0.3	26 ± 0.4	24 ± 0.3
10	Tetracyclines	Doxycycline	12 ± 0.2	18 ± 0.4	12 ± 0.2
11	Phenicols	Florfenicol	18 ± 0.3	24 ± 0.4	18 ± 0.3
12	Sulfonamides	Sulfamonomethoxine	10 ± 0.2	16 ± 0.4	10 ± 0.2
13	Polypeptides	Colistin	16 ± 0.3	20 ± 0.3	18 ± 0.3
14	Macrolides	Tilmicosin	0 ± 0	10 ± 0.3	10 ± 0.3
15	Others	Mequindox	0 ± 0	16 ± 0.4	10 ± 0.3

### 3.5 The *gltA* deletion reduces *S*. Enteritidis stress tolerance

**Figure 4:**
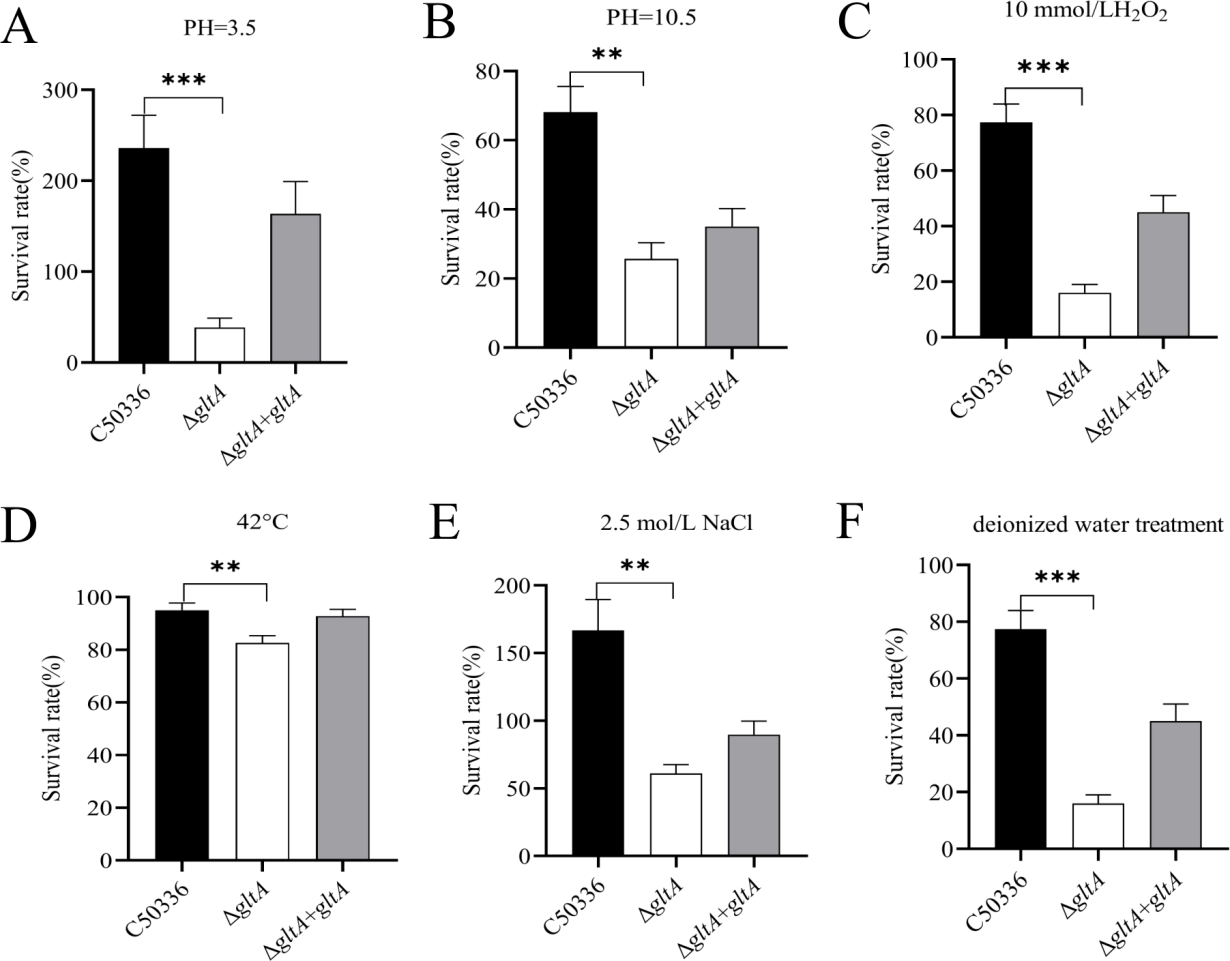
Survival rates of Δ*gltA* under environmental stresses. Mid-logarithmic-phase bacterial cultures were standardized to an initial concentration of 1 × 10^7^ CFU/mL in sterile physiological saline. For prolonged abiotic stress assays, standardized bacterial suspensions were diluted at a 1:100 ratio and incubated for 1 h under five independent stress conditions: **(A)** Acidic stress, **(B)** Alkaline stress, **(D)** Heat stress, **(E)** Hyperosmotic stress, **(F)** hypoosmotic stress. For **(C)** acute Oxidative stress, bacterial pellets were resuspended in PBS containing 10 mmol/L H_2_O_2_ and incubated for 10 min at 37 °C, with shorter incubation duration adopted to avoid rapid bacterial lysis. Following all stress treatments, cell suspensions were serially ten-fold diluted in sterile PBS, and viable bacterial colonies were enumerated via plate counting. Bacterial survival rate was defined as the percentage of viable CFU recovered post-stress relative to pre-stress baseline CFU counts. Data are presented as mean values of three replicates. Data are presented as mean ± standard error of the mean (SEM) from three independent experiments. Statistical significance was determined by Student’s t-test for comparisons between two groups. (**p < 0.01, ***p < 0.001).

Bacterial survival was quantified under acidic (pH 3.5), alkaline (pH 10.5), oxidative (10 mmol/L H_2_O_2_), heat (42 °C), hyperosmotic (2.5 mol/L NaCl), and hypoosmotic stress conditions. Δ*gltA* exhibited significantly reduced survival under all tested stresses compared with C50336 (**Figure 4**). Survival rates of Δ*gltA* were only 15%–20% of the wild-type under severe stresses (pH 3.5, H_2_O_2_, hypoosmotic stress; ***p < 0.001), and were also markedly reduced under heat, alkaline, and hyperosmotic stresses (**p < 0.01). Stress resistance was greatly restored in the complemented strain. These data establish that GltA is essential for *S*. Enteritidis to tolerate diverse environmental stresses.

### 3.6 The *gltA* deletion downregulates virulence gene expression in *S*. Enteritidis

**Figure 5:**
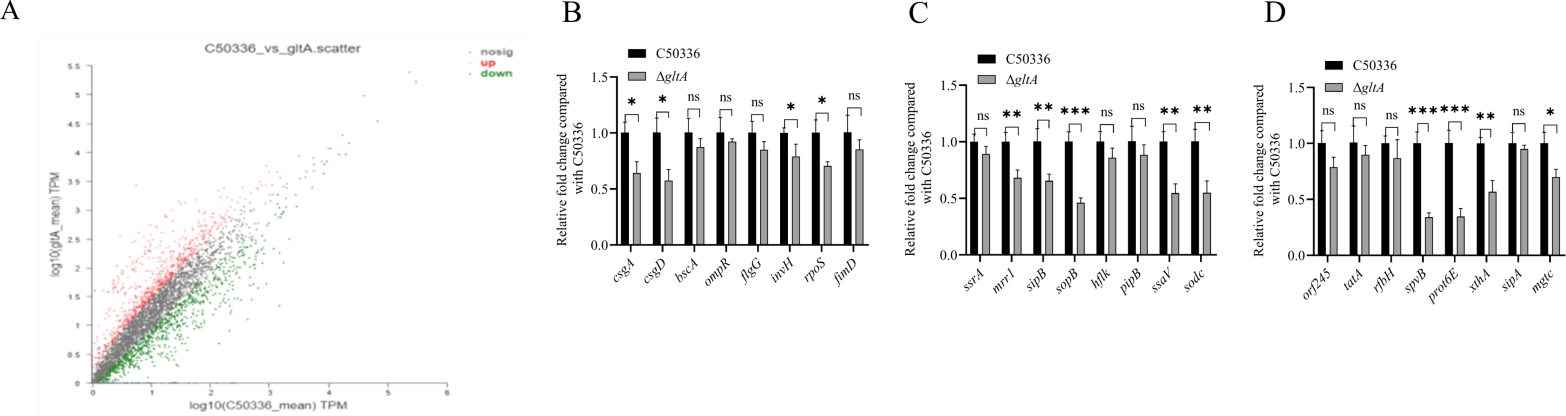
Transcriptome profiling and virulence gene expression analysis. **(A)** C50336 and Δ*gltA* strains were cultured in shaking LB broth until OD_600_ reached 1.0–2.0. Cell pellets were washed three times with sterile PBS, snap-frozen in liquid nitrogen and stored at −80 °C. After extracting bacterial RNA, the samples were sent to Shanghai Majorbio Bio-Pharm Technology Co., Ltd. for transcriptomic sequencing analysis using the Illumina NovaSeq platform. **(B–D)** qRT-PCR analysis of virulence gene expression. The expression levels of the virulence genes in C50336, Δ*gltA*, and Δ*gltA* + *gltA* were measured by qPCR using 16S rRNA as an internal control. Data are presented as mean values of three replicates. Data are presented as mean ± standard error of the mean (SEM) from three independent experiments. Statistical significance was determined by Student’s t-test for comparisons between two groups. (*p < 0.05, **p < 0.01, ***p < 0.001).

RNA sequencing (RNA-Seq) was performed to profile global transcriptional changes induced by *gltA* deletion. A total of 1184 differentially expressed genes (DEGs) were identified, including 594 upregulated and 590 downregulated genes (**Figure 5A**). GO functional annotation and KEGG pathway enrichment analyses demonstrated that deletion of the Δ*gltA* gene exerts regulatory effects on multiple biological processes in *Salmonella* Enteritidis (**Supplementary Figures 1**, **S2**). PCA exhibited tight intra-group clustering for both C50336 and Δ*gltA* samples, confirming strong reproducibility of biological replicates (**Supplementary Figure 3**). This balanced transcriptional alteration suggests that *gltA* exerts bidirectional regulatory effects on *S*. Enteritidis gene expression. Importantly, these transcriptomic associations require independent functional validation to confirm causal regulatory relationships between *gltA* deletion and altered virulence gene transcription.

qRT-PCR was used to validate the expression of 24 key virulence genes. The *gltA* deletion resulted in widespread downregulation of genes associated with biofilm synthesis (*csgA*, *csgD*, *flgG*), type III secretion systems, virulence plasmids, and stress regulation (*mrr1*, *sopB*, *sodC*) (**Figures 5B, D**). These transcriptional changes are consistent with the impaired motility, biofilm formation, and stress tolerance phenotypes, validating the RNA-Seq data and identifying key molecular targets underlying *gltA*-mediated virulence regulation.

### 3.7 The *gltA* deletion drastically attenuates *S*. Enteritidis virulence *in vivo*

Bacterial virulence was assessed in 6–8-week-old female Kunming mice via intraperitoneal infection. The LD_50_ of wild-type C50336 was 3.16 × 10³ CFU, indicating high virulence. In contrast, no mortality was observed in mice challenged with Δ*gltA* at the maximum dose of 1 × 10^9^ CFU, representing an over 10^6^-fold attenuation of virulence (**Table 4**).

**Table 4:** LD_50_ values of C50336 and Δ*gltA* in Kunming mice.

Strain	Inoculation dose (CFU/mouse)	No. of deaths/total no. of mice	LD_50_ (CFU)
C50336	1×10^5^	5/5	3.16×10^3^
	1×10^6^	5/5	
	1×10^7^	5/5	
	1×10^8^	5/5	
	1×10^9^	5/5	
Δ*gltA*	1×10^5^	0/5	
	1×10^6^	0/5	
	1×10^7^	0/5	
	1×10^8^	0/5	
	1×10^9^	0/5	

**Figure 6:**
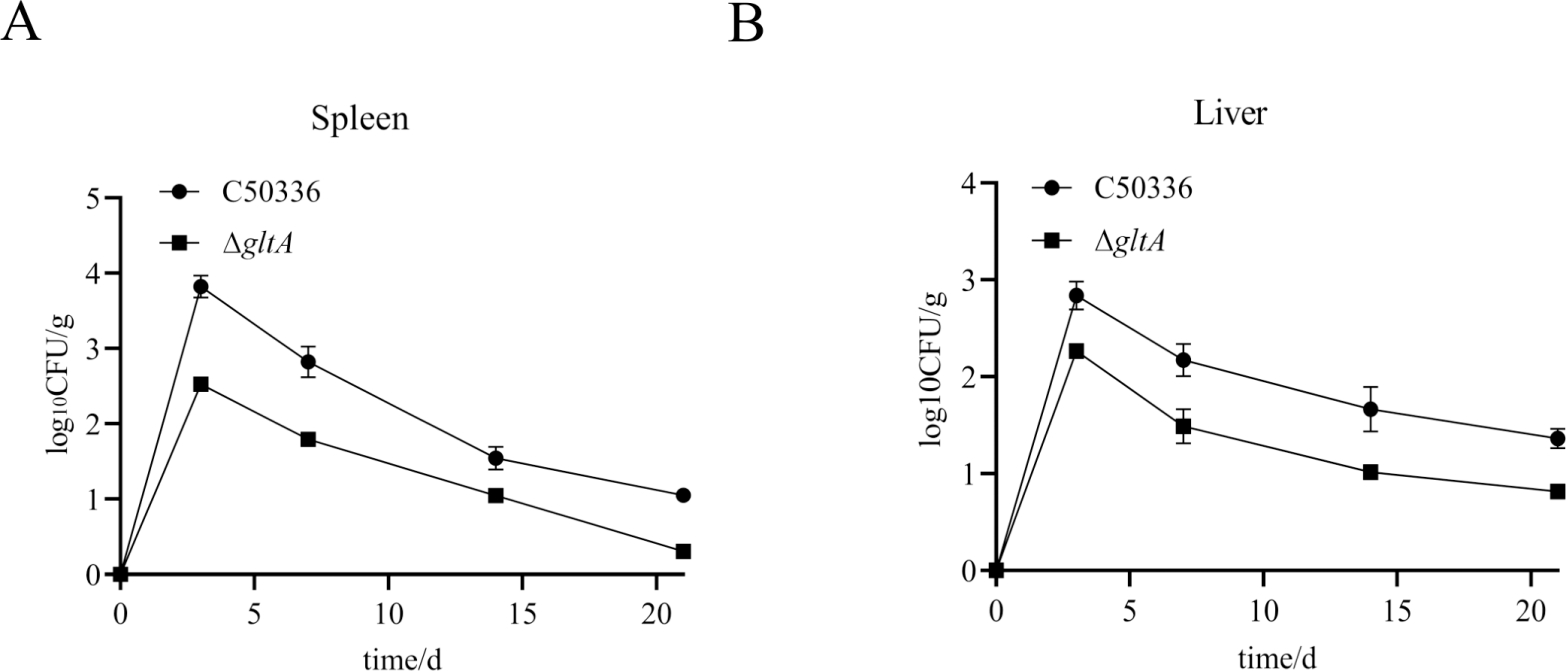
Bacterial loads in mouse tissues post-infection. **(A)** Twenty female/male mice were randomly allocated into two independent groups (n=10 for each group). Each mouse was challenged with 1 × 10^4^ CFU of wild-type C50336 or Δ*gltA* strain via routine inoculation. Mice were euthanized, and spleen tissues were aseptically harvested at 7, 14, 21 and 28 days post-infection. Isolated tissues were weighed individually and homogenized in pre-chilled sterile PBS. Tissue homogenates were serially ten-fold diluted with sterile PBS, and diluted suspensions were spread on selective agar plates for viable bacterial enumeration. **(B)** Twenty female/male mice were randomly allocated into two independent groups (n=10 for each group). Each mouse was challenged with 1 × 10^4^ CFU of wild-type C50336 or Δ*gltA* strain via routine inoculation. Mice were euthanized, and liver tissues were aseptically harvested at 7, 14, 21 and 28 days post-infection. Isolated tissues were weighed individually and homogenized in pre-chilled sterile PBS. Tissue homogenates were serially ten-fold diluted with sterile PBS, and diluted suspensions were spread on selective agar plates for viable bacterial enumeration. Data are presented as mean values of three replicates. Twenty female/male mice were randomly allocated into two independent groups (n=10 for each group).

Bacterial loads in the liver and spleen were quantified at multiple time points post-infection. Δ*gltA* exhibited significantly lower bacterial burdens in both organs at all time points compared with C50336 (**Figures 6A, B**). These results confirm that *gltA* is critical for *S*. Enteritidis *in vivo* colonization and pathogenicity in mice.

### 3.8 Δ*gltA* confers robust immunoprotection against *S*. Enteritidis challenge

**Figure 7:**
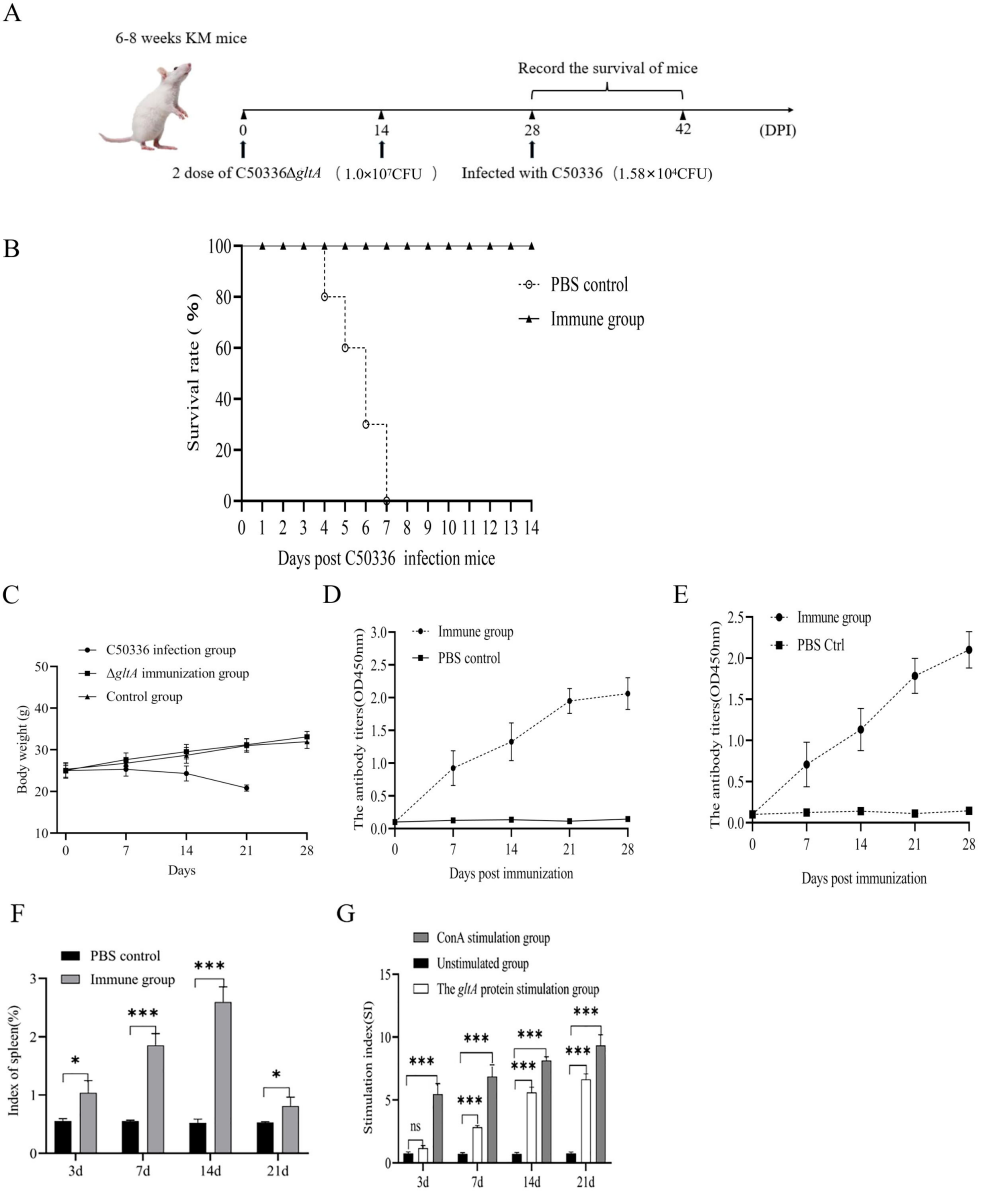
Evaluation of immunoprotective efficacy of the Δ*gltA* mutant. **(A)** Kunming mice were immunized with Δ*gltA* for 28 days and infected with C50336 by intraperitoneal injection. Mice were observed daily for mortality. **(B)** Forty Kunming mice were randomly divided into two groups (n = 20 per group). Mice in the immunization group were orally inoculated with 1.0 × 10^7^ CFU of the Δ*gltA* strain, while mice in the control group were orally administered an equal volume of sterile PBS. After 28 days, all mice were challenged intraperitoneally with a dose of 1.58 × 10^4^ CFU of the wild-type C50336 strain. For 14 consecutive days following the challenge, mouse mortality was monitored and recorded daily. **(C)** Changes in body weight in mice following immunization with Δ*gltA* strain. **(D)** Serum IgG levels analysis. Kunming mice were orally administered with the Δ*gltA* strain, and the serum IgG antibody levels were measured by ELISA on days 7, 14, 21, and 28 post-immunization. **(E)** Serum sIgA levels analysis. Kunming mice were orally administered with the Δ*gltA* strain, and the serum sIgA antibody levels were measured by ELISA on days 7, 14, 21, and 28 post-immunization. **(F)** Spleen indices post-immunization. Three mice from each group were randomly selected and weighed at 7, 14, 21 and 28 days post-immunization. Selected mice were humanely euthanized via gradual CO_2_ inhalation (chamber volume displacement rate: 30%-40% per minute). Spleens were aseptically excised and weighed post-euthanasia. The spleen index was calculated using the formula: (spleen weight/body weight) × 100%. **(G)** Splenocyte proliferation assay. Kunming mice (n=20 per group) received oral immunization with 1.0 × 10^7^ CFU Δ*gltA* or PBS. Three mice per group were euthanized periodically for spleen index measurement, and another three were sampled on day 14 post-immunization for splenocyte isolation. Splenocytes were purified via 70 μm filtration and erythrocyte lysis, then cultured in complete RPMI 1640 medium. Isolated cells were stimulated with bacterial supernatant antigen, concanavalin A or PBS for 72 h at 37 °C with 5% CO_2_. Lymphocyte proliferation was detected by MTT assay, with stimulation index calculated as: SI = (OD_450_ of stimulated group − OD_450_ of medium-only group)/(OD_450_ of unstimulated group − OD_450_ of medium-only group). Data are presented as mean values of three replicates. Data are presented as mean ± standard error of the mean (SEM) from three independent experiments. Statistical significance was determined by Student’s t-test for comparisons between two groups. (ns means not significant, *p < 0.05, ***p < 0.001).

The mice were divided into two groups for oral immunization: one group was inoculated with Δ*gltA*, while the other group was gavaged with an equal volume of PBS as a control. On day 28 post-immunization, the mice underwent an intraperitoneal challenge with wild-type C50336 to evaluate the immunoprotective effect (**Figure 7A**). Daily clinical monitoring was performed on all immunized mice. All immunized mice survived at a 100% rate for 14 days post-challenge. in contrast, all unimmunized mice exhibited clinical symptoms such as loss of appetite, reduced activity, and rough fur, and all died within 7 days of challenge, demonstrating excellent immunoprotective effects (**Figure 7B**). Body weight monitoring showed comparable weight trajectories in Δ*gltA*-immunized and PBS control mice. Mice infected with wild-type C50336 displayed progressive weight loss, with average body weight falling to ~20 g at day 21, when these animals were humanely euthanized. (**Figure 7C**).

Serological analysis showed a continuous increase in *S*. Enteritidis-specific IgG levels in immunized mice from day 7 to 28 post-immunization, indicating a robust humoral immune response (**Figure 7D**). SIgA antibody testing revealed that fecal sIgA levels remained elevated between days 7 and 28 post-vaccination and were significantly higher than those in the control group (**Figure 7E**). This indicates that Δ*gltA* can induce a mucosal immune response in mice.

Immunized mice also had significantly higher spleen indices than controls (**Figure 7F**). Lymphocyte proliferation assays revealed significantly elevated stimulation indices in immunized mice following bacterial antigen stimulation, demonstrating strong cellular immune activation (**Figure 7G**). Collectively, these data demonstrate that Δ*gltA* immunization induces measurable humoral, mucosal and cellular immune responses in mice and delivers full survival against lethal wild-type challenge within this mouse intraperitoneal challenge model.

## 4 Discussion

This study demonstrates that deletion of the TCA cycle rate-limiting enzyme gene *gltA* generates a mutant strain with over 10^6^-fold attenuated virulence in *S*. Enteritidis while preserving normal growth and robust immunogenicity in mice. These findings establish a novel link between central carbon metabolism and pathogenicity in *S*. Enteritidis and identify *gltA* as a promising target for live attenuated vaccine development.

In this study, Δ*gltA* showed identical growth rates to the wild-type C50336 strain in both nutrient-rich LB medium and nutrient-deficient M9 minimal medium. This phenotype contrasts sharply with prior observations in *Escherichia coli* (17) and *Klebsiella pneumoniae* (16), in which loss of *gltA* abrogates bacterial proliferation in minimal medium. Previous studies have confirmed that citrate synthase, as the rate-limiting enzyme at the start of the TCA cycle, when functionally deficient, directly blocks the central carbon metabolism pathway in most pathogenic bacteria, rendering the strains unable to survive in minimal media without exogenous citrate supplementation. However, in this study, *S*. Enteritidis maintained normal growth even after GltA deletion. It is speculated that *S*. Enteritidis may possess alternative carbon metabolism pathways (such as the glyoxylate shunt or acetone cycle) that compensate for TCA cycle dysfunction. Targeted metabolomics analysis will be required to identify the exact compensatory mechanism (24). This phenotype holds substantial practical value for the industrial development of attenuated vaccines. Unlike classic auxotrophic attenuation targets such as *aroA* and *asd*, the Δ*gltA* mutant can achieve high-density *in vitro* culture without adding specific amino acids or metabolic substrates to the medium, thereby significantly reducing the technical complexity and economic costs associated with large-scale vaccine production.

Motility and biofilm formation are core biological traits for *S*. Enteritidis to achieve intestinal adhesion, colonization, and immune evasion in the host. In this study, we found that *gltA* deletion significantly reduced the motility diameter of *S*. Enteritidis on semi-solid medium. Crystal violet staining, Congo red staining, and Calcofluor white staining confirmed that Δ*gltA* had significantly impaired biofilm formation ability. The synthesis of core biofilm components, Curli fimbriae and cellulose, was markedly inhibited. All these phenotypes were restored by complementation with wild-type *gltA*. These results are highly consistent with previous studies in *E. coli* and Bacillus subtilis, where *gltA* deletion significantly inhibited bacterial biofilm formation (25). It is important to note that these visual assessments remain purely qualitative without supporting quantitative measurements or statistical comparison. The reliance on representative micrographs limits our capacity to precisely quantify strain-specific differences in colony texture and biofilm formation. Future work incorporating morphometric quantification and statistical analysis of surface architecture will be required to further validate the regulatory role of *gltA* in modulating extracellular matrix biogenesis. In addition, biofilm matrices also act as a physical barrier that hinders antibiotic permeation and elevates intrinsic bacterial antimicrobial tolerance. The compromised biofilm production observed in the Δ*gltA* mutant may thus partially explain its enhanced susceptibility to antimicrobials. However, additional mechanisms such as altered energy metabolism and reduced expression of efflux pumps cannot be ruled out and warrant further investigation. In particular, susceptibility to tilmicosin and mequindox—to which the wild-type strain was intrinsically susceptible—was significantly restored in the deletion mutant. This finding suggests that *gltA* can serve as a novel target for antimicrobial adjuvant development.

*In vivo* infection by *S*. Enteritidis relies on sequential bacterial evasion of host physical barriers, adaptation to diverse microenvironmental stresses, and sustained intratissular colonization and replication. Stress survival assays demonstrated that loss of *gltA* markedly impaired the tolerance of *S*. Enteritidis to a broad panel of stressors, including acid, alkali, oxidative insult, high temperature, hyperosmolarity and hypoosmolarity. These results support prior evidence linking an intact TCA cycle to bacterial stress resistance (26, 27). Acid tolerance enables *S*. Enteritidis to traverse the gastric acid barrier and colonize the gut, whereas oxidative stress resilience governs bacterial persistence against the macrophage respiratory burst—a critical step for systemic dissemination (28–30). Under pH 3.5 acid challenge and 10 mM H_2_O_2_ oxidative exposure, the Δ*gltA* mutant exhibited drastically lower survival relative to wild-type C50336. This impaired stress adaptation severely restricts its persistence within intestinal epithelia and macrophages, forming a primary mechanistic basis for its profound virulence attenuation. Mouse lethal dose assays yielded an intraperitoneal LD_50_ of 3.16 × 10³ CFU for wild-type C50336 in Kunming mice. By contrast, no mortality occurred in animals challenged with the Δ*gltA* mutant even at the maximum inoculum of 1 × 10^9^ CFU, corresponding to a >10^6^-fold reduction in virulence relative to the parental strain. The magnitude of this attenuation matches that of *S*. Enteritidis attenuated mutants generated via canonical vaccine targets including *aroA* and *crp* (31, 32). Consistent with these lethal dose data, organ bacterial load quantification revealed substantially impaired colonization and replication of the Δ*gltA* mutant within mouse liver and spleen tissues. A limitation of this organ load assay is that we did not quantify bacterial colonization within intestinal tissues, the primary natural infection site of *S*. Enteritidis. The Δ*gltA* mutant was almost completely cleared by the host immune system at 21 days post-infection, further validating its significantly attenuated virulence phenotype. Consistent with our transcriptomic profiling and qRT-PCR validation, Δ*gltA* deletion correlates with global downregulation of core SPI-1 and SPI-2 virulence genes, though this correlative link requires further functional testing to confirm direct regulatory control. Numerous previous studies have confirmed that the type III secretion system (T3SS) encoded by SPI-1 mediates *S*. Enteritidis invasion of host intestinal epithelial cells. SPI-2 regulates bacterial survival and replication in macrophages. These two pathogenicity islands are the core determinants of *S*. Enteritidis pathogenicity (33, 34). These results indicate that *gltA* not only indirectly affects bacterial virulence by regulating metabolism and environmental adaptability, but also participates in *S*. Enteritidis pathogenesis by regulating the expression of SPI genes.

Based on our transcriptomic findings and established knowledge of TCA cycle-mediated virulence regulation, we propose a hypothetical model where Δ*gltA* deletion modulates virulence via altering intracellular pools of TCA intermediates. Citrate synthase serves as a rate-limiting enzyme of the tricarboxylic acid cycle. Deletion of its encoding gene may trigger profound alterations in the intracellular abundances of central TCA metabolites, including acetyl-CoA, citrate, α-ketoglutarate, and succinate. Numerous metabolites within this pool—such as α-ketoglutarate and succinate—function as signaling molecules that directly modulate the activities of virulence-associated transcriptional regulators, exemplified by the two-component system KguS/KguR (35), the sigma factor RpoS, citrate, and cyclic adenosine monophosphate (cAMP); these signaling cues are tightly coupled to *Salmonella* infection progression. For instance, α-ketoglutarate modulates *Pseudomonas aeruginosa* virulence via targeting the KguS/KguR two-component regulatory system (36, 37), while succinate acts as a critical signal governing virulence regulation during intracellular *Salmonella* infection. Accordingly, we hypothesize *gltA* deletion exerts regulatory effects indirectly by perturbing downstream metabolite pools. Nevertheless, definitive experimental validation is required to pinpoint the principal metabolite mediating the phenotypic outcomes of *gltA* deletion.

The core challenge in developing live attenuated vaccines lies in achieving a precise balance between biosafety and immunogenicity. Insufficient attenuation can lead to residual virulence in vaccines, posing safety risks for clinical application, while excessive attenuation can result in reduced immunogenicity, failing to induce an effective protective immune response in the host. The results of this study show that although the Δ*gltA* strain exhibits more than 10^6^-fold reduction in virulence and possesses extremely high biosafety, it retains good immunogenicity. Oral immunization of Kunming mice with Δ*gltA* induced a continuous increase in serum *S*. Enteritidis-specific IgG antibody levels and sIgA level in feces. Simultaneously, the spleen index and specific proliferative capacity of splenic lymphocytes in mice were significantly enhanced, confirming that this deletion strain can activate both humoral and cellular immune responses in the host. Of course, our immunological assessment is limited to antibody ELISA (serum IgG, fecal sIgA) and splenocyte proliferation assays. Functional immune tests including serum bactericidal assay (SBA) and opsonophagocytic killing (OPK), as well as antigen-specific T cell cytokine profiling, were not performed in the present study, restricting our ability to evaluate the full functional potency of the induced immune response, and these works will be addressed in future research. Results from the lethal dose challenge revealed that a single oral immunization with 1×10^7^ CFU of Δ*gltA* strain conferred robust protection mice with a survival rate of 100%. All mice from the immunized group survived 14 days post-challenge without obvious clinical symptoms, demonstrating significantly superior protective efficacy compared to most previously reported *S*. Enteritidis attenuated vaccine candidates (38, 39). Unlike inactivated vaccines, which only induce humoral immunity and fail to activate cellular and mucosal immunity, live attenuated vaccines can mimic the natural infection process of *S*. Enteritidis via the oral route, simultaneously activating the host’s mucosal, humoral, and cellular immunity, which may be the core reason for their effective immunoprotection. In this study, although the colonization ability of Δ*gltA* in host organs was significantly lower than that of the wild-type strain, it still achieved limited proliferation and colonization *in vivo*, allowing continuous stimulation of the host immune system, thereby inducing a comprehensive and long-lasting immune response, which perfectly matches the core characteristics of an ideal live attenuated vaccine.

Although this study systematically elucidated the biological function of *gltA* in *S*. Enteritidis and its potential for use in attenuated vaccines, there are still some limitations that require further investigation and validation in subsequent studies. First, this study only evaluated the attenuation effect and immunoprotective efficacy of the deletion mutant in a mouse model. The challenge via intraperitoneal injection is not the natural route of infection, thus may not accurately reflect the immunoprotective efficacy of the *gltA*-deficient strain. Systematic *in vivo* tests have not been carried out in poultry, the natural host of *S*. Enteritidis. Poultry serve as the primary reservoir host and transmission vector of *S*. Enteritidis, and are also the core target animal for *Salmonella* vaccine prevention and control. Significant differences exist between mice and poultry in terms of immune system composition, intestinal microenvironment, and pathogen colonization patterns. A chicken model is required to further evaluate the oral safety, *in vivo* colonization and clearance patterns, immunoprotective efficacy, and ability of this deletion strain to block vertical transmission in hatching eggs, thereby providing core data support for its clinical application in livestock and poultry farming. Secondly, although this study employed transcriptome sequencing to clarify the changes in the whole-genome expression profile of *S*. Enteritidis following *gltA* deletion and confirmed that it affects the expression of genes related to motility, biofilm formation, and virulence, the specific molecular mechanisms underlying its regulatory role have not yet been thoroughly elucidated. It remains to be clarified how the metabolic signals triggered by *gltA* deletion are transmitted to downstream target genes via global regulatory factors, and the downstream signaling pathways of *gltA* require further clarification. Additionally, this study did not systematically evaluate the long-term genetic stability and environmental biosafety risks of the Δ*gltA* mutant. Although we have previously serially passaged the Δ*gltA* mutant *in vitro* for 50 generations without observing gene reversion, we have not yet completed verification through additional generations or *in vivo* back-passage tests.

All phenotypic experiments including growth, motility, biofilm, stress resistance, immunological assays were performed with n=3 biological replicates, except for animal infection groups (n=5 or n=10 per group). Small replicate numbers may reduce overall statistical power, and multiple testing corrections were not applied to individual phenotypic comparisons; only RNA-seq utilized adjusted padj (FDR) values to control false positives. Follow-up experiments with larger sample sizes will help confirm the robustness of these phenotypic differences.

Taken together, the present study demonstrates that *gltA* is a key functional gene associated with environmental adaptability and pathogenicity of *S*. Enteritidis. The Δ*gltA* mutant exhibits excellent attenuation and immunoprotective efficacy. These findings provide new insights into *S*. Enteritidis pathogenesis and promising targets for *Salmonella* vaccine development.

## Data Availability

The data that support the findings of this study are openly available in Science Data Bank at https://www.scidb.cn/s/Arem22. The DOI is 10.57760/sciencedb.35201.
