# Supplementary material for: Deletion of gltA attenuates virulence and confers immune protection against Salmonella Enteritidis
Source: Front Immunol. 2026 Jul 15;17:1869123. doi: 10.3389/fimmu.2026.1869123 (PMC13414736; doi:10.3389/fimmu.2026.1869123)
Supplement: Supplementary file 1 [file DataSheet1.pdf]

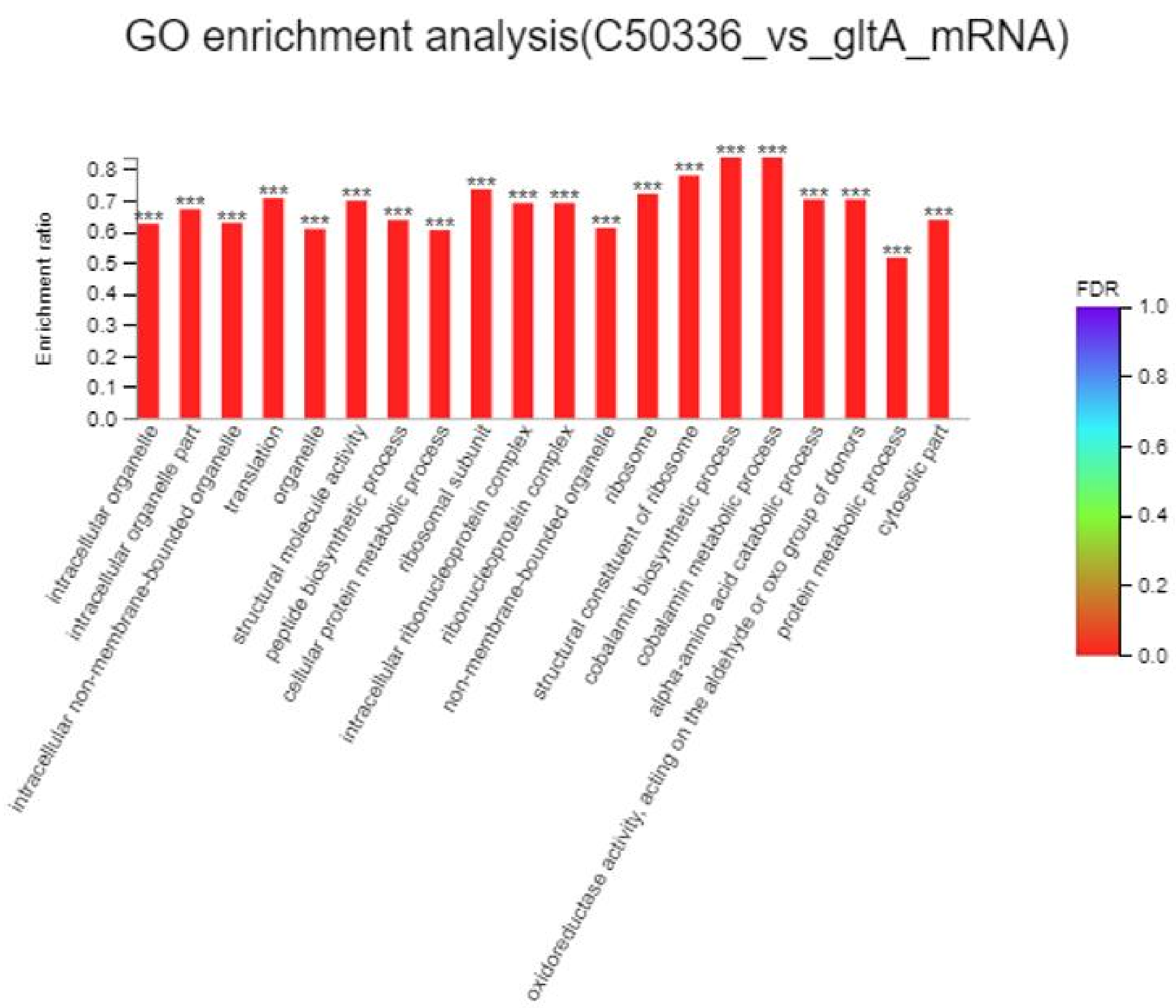

Fig. S1: Top enriched GO terms of DEGs categorized into biological process and cellular component.
