# Supplementary material for: Deletion of gltA attenuates virulence and confers immune protection against Salmonella Enteritidis
Source: Front Immunol. 2026 Jul 15;17:1869123. doi: 10.3389/fimmu.2026.1869123 (PMC13414736; doi:10.3389/fimmu.2026.1869123)
Supplement: Supplementary file 2 [file DataSheet2.pdf]

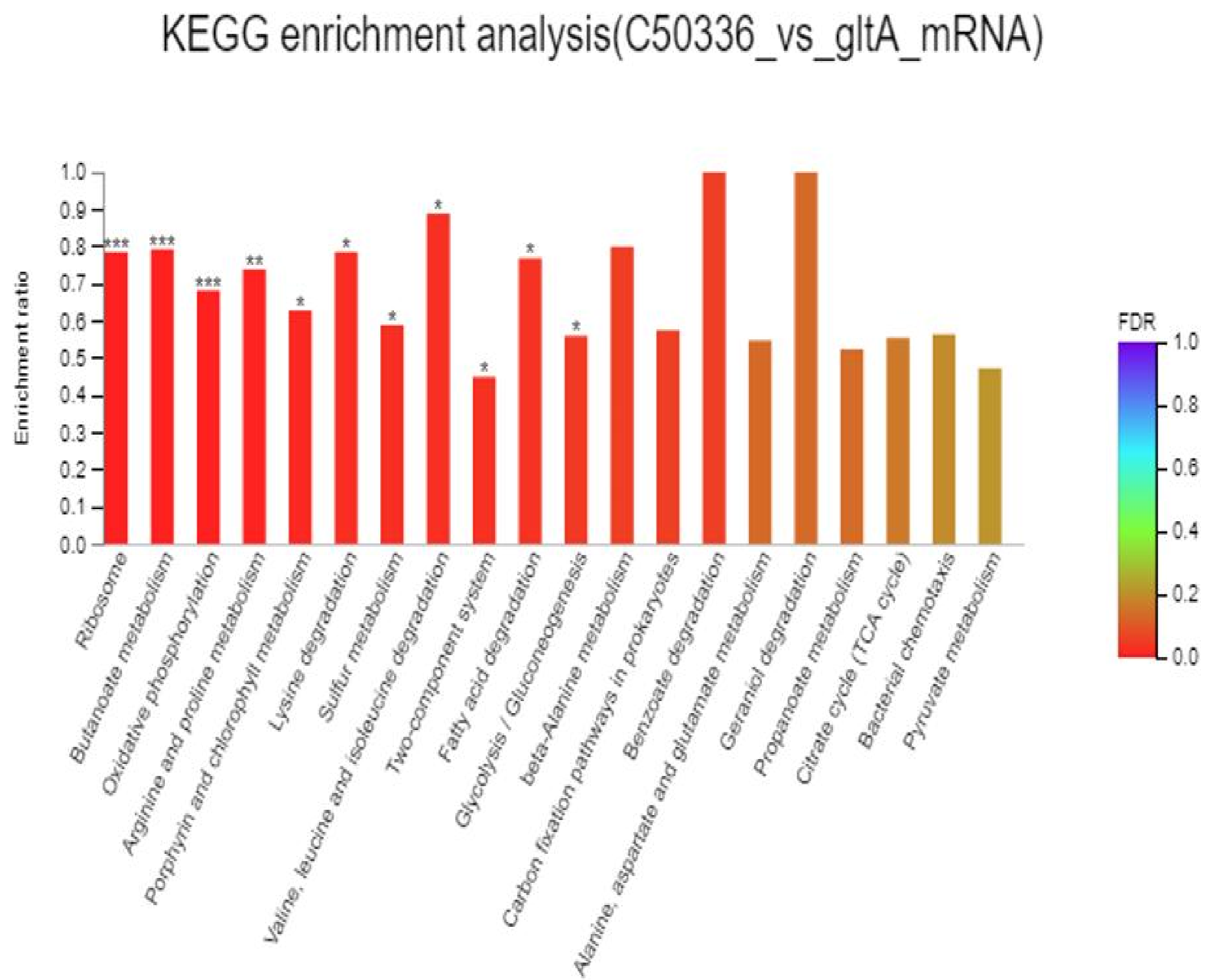

Fig. S2: Top enriched KEGG pathways. The horizontal axis represents enrichment ratio, and asterisks indicate the significance of FDR-adjusted P-value. Pathways marked without asterisk show enrichment tendency but no statistically significant difference.
