# Supplementary material for: Deletion of gltA attenuates virulence and confers immune protection against Salmonella Enteritidis
Source: Front Immunol. 2026 Jul 15;17:1869123. doi: 10.3389/fimmu.2026.1869123 (PMC13414736; doi:10.3389/fimmu.2026.1869123)
Supplement: Supplementary file 3 [file DataSheet3.pdf]

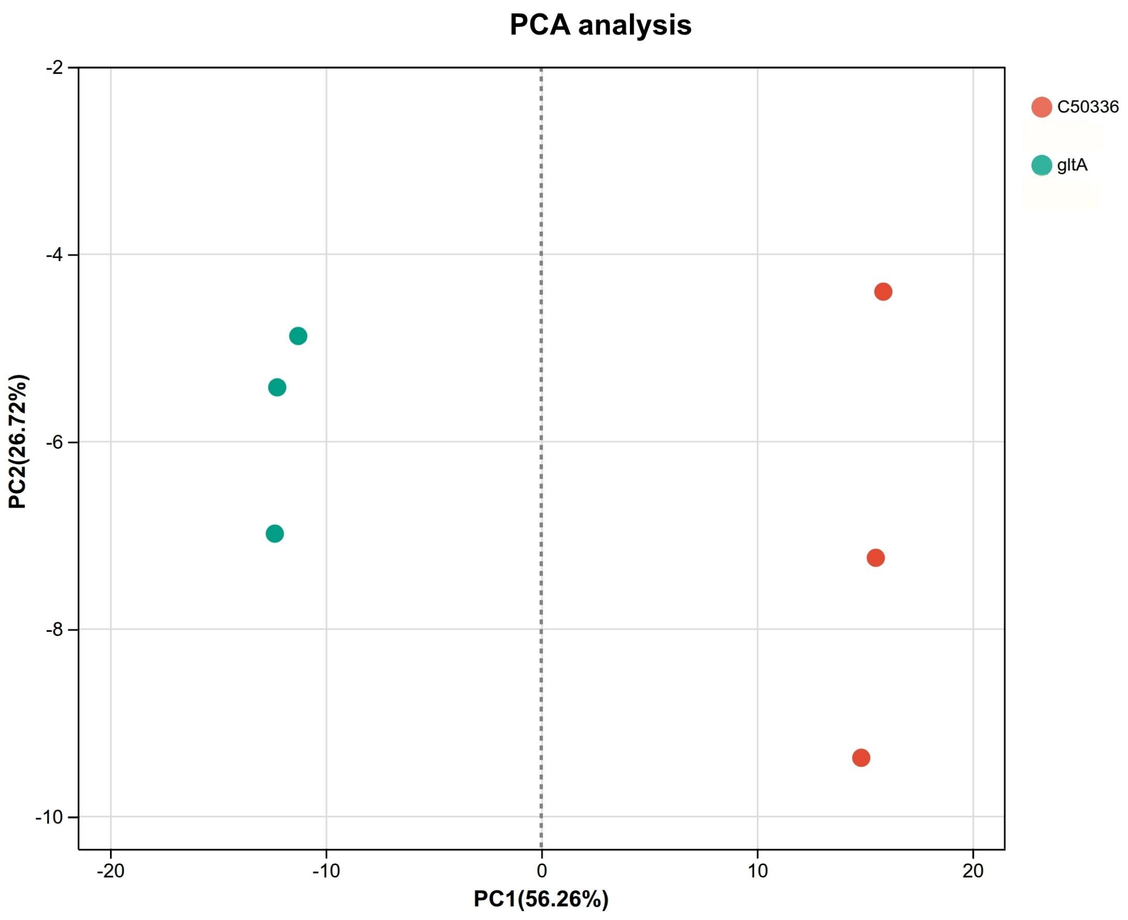

Fig. S3: Principal component analysis of transcriptomic data from wild-type C50336 and Δ*gltA*. PCA was used to assess sample similarity based on normalized gene expression levels.
